# Effects of High-Humidity Aging on Platinum, Palladium, and Gold Loaded Tin Oxide—Volatile Organic Compound Sensors

**DOI:** 10.3390/s100706513

**Published:** 2010-07-06

**Authors:** Toshio Itoh, Ichiro Matsubara, Masahiro Kadosaki, Yuichi Sakai, Woosuck Shin, Noriya Izu, Maiko Nishibori

**Affiliations:** 1 National Institute of Advanced Industrial Science and Technology (AIST), Shimo-Shidami, Moriyama-ku, Nagoya 463-8560, Japan; E-Mails: matsubara-i@aist.go.jp (I.M.); w.shin@aist.go.jp (W.S.); n-izu@aist.go.jp (N.I.); m-nishibori@aist.go.jp (M.N.); 2 Toyama Industrial Technology Center, 383 Takada, Toyama 930-0866, Japan; E-Mails: kado@itc.pref.toyama.jp (M.K.); sakai@itc.pref.toyama.jp (Y.S.)

**Keywords:** gas sensor, tin oxide, noble metal loaded tin oxide, T–VOC test gas, high-humidity aging

## Abstract

This study is an investigation of high-humidity aging effects on the total volatile organic compound (T–VOC) gas-sensing properties of platinum, palladium, and gold-loaded tin oxide (Pt,Pd,Au/SnO_2_) thick films. The sensor responses of the high-humidity aged Pt,Pd,Au/SnO_2_, a non-aged Pt,Pd,Au/SnO_2_, and a high-humidity aged Pt/SnO_2_ to T–VOC test gas have been measured. The high-humidity aging is an effective treatment for resistance to humidity change for the Pt,Pd,Au/SnO_2_ but not effective for the Pt/SnO_2_. The mechanism of the high-humidity aging effects is discussed based on the change of surface state of the SnO_2_ particles.

## 1. Introduction

Sick building syndrome is caused by harmful volatile organic compounds (VOCs), even where the concentration of VOCs is low, *i.e.*, ppb level. Formaldehyde, one of the harmful VOCs, is widely known to be an important cause of sick building syndrome. Numerous investigations have been carried out for the development of high-sensitivity formaldehyde sensors [1–4]. However, the sick building syndrome is caused not only by formaldehyde but also by other harmful chemicals. Moreover, the use of harmful chemicals has been restricted, but alternative chemicals, whose risks to human health are poorly understood, tend to be used in their place. It is, therefore, desirable that we use high sensitivity VOC sensors and those be able to detect all VOCs, *i.e.*, a T–VOC sensor.

Metal oxides are one of the best materials for VOC sensors because of the simplicity of the gas sensor system. We have previously reported on high sensitivity SnO_2_-based gas sensors as T–VOC gas sensors [5–7]. The SnO_2_-based gas sensors showed lower response to aliphatic hydrocarbons, halogenated hydrocarbons, and aromatic hydrocarbons than to other groups of VOCs. It has been reported that the addition of Pt, Pd, and Au to SnO_2_ thick films to improve the sensitivity to aliphatic, halogenated, and aromatic hydrocarbons, respectively, is effective [6]. Interestingly, all of these noble metal-loaded SnO_2_ (Pt,Pd,Au/SnO_2_) sensors have good performance, not only in their response to several VOCs but also in the immunity of the sensor-response to humidity [7]. However, further improvement of the immunity to humidity change is required, especially for use in high humidity regions. In this study, we have investigated on the effects of high-humidity aging on the responsitivity of the Pt, Pd, Au/SnO_2_ thick film elements to improve their immunity to humidity changes.

## 2. Experimental

### 2.1. Synthesis of Pt,Pd,Au/SnO_2_ and Pt/SnO_2_ thick films

The Pt,Pd,Au/SnO_2_ and Pt/SnO_2_ thick film elements were prepared according to our previous report [5–7]. Pt, Pd, and Au colloid suspensions (Toda Kogyo Corp.; particle size: 2–5 nm) were added to the SnO_2_ powder (C.I. Kasei NanoTec; std. particle size: 20–30 nm). The contents of Pt, Pd, and Au were 0.5, 0.8, and 0.5 wt% relative to that of the SnO_2_, respectively. The mixture was stirred and dried at 120 °C. In the case of Pt/SnO_2_, only 0.5 wt% of Pt was added to the SnO_2_ powder. The resulting Pt,Pd,Au-added SnO_2_ (Pt,Pd,Au/SnO_2_) and Pt-added SnO_2_ (Pt/SnO_2_) powders were ground, and added into an ethyl cellulose based vehicle to obtain Pt,Pd,Au/SnO_2_ and Pt/SnO_2_ pastes. The sensor elements were prepared by a screen printing method. The pastes were printed on alumina substrates with a pair of gold parallel electrodes with a gap of 0.5 mm. The Pt,Pd,Au/SnO_2_ and Pt/SnO_2_–printed substrates were heated at 600 °C for 1 h.

### 2.2. Aging

The high-humidity, room air, and dry air agings of the elements were carried out using a flow-type apparatus. The elements were placed into quartz tube with an inside diameter of 25 mm. Humid air, with a relative humidity of up to 90% at room temperature (RT), or dry synthetic air was flowed at a rate of 250 mL/min. In a case of room air aging, both ends of the quartz tube were opened. The quartz tube was placed in an electric furnace and heated to 400 °C for approximately two weeks. After the aging, the elements were cooled to RT and subsequently set into a gas sensing measurement apparatus.

### 2.3. Gas sensing properties of Pt,Pd,Au/SnO_2_

The molar ratios of each VOC component in the T–VOC test gas was determined on the basis of recent chemical analysis data on the indoor air condition of Japanese residences, which was reported by Osawa *et al.* [8]. Table 1 shows the concentrations of each VOC component in the T–VOC test gas (Sumitomo Seika Chemicals). The balance gas of the T–VOC test gas was nitrogen. The total concentration of the T–VOC gas was 10.29 ppm, *i.e.*, 3.66 × 10^4^ μg/m^3^ [7].

**Figure 1.**
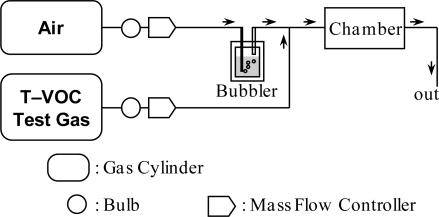
Schematic diagram of a flow-type gas sensing measurement apparatus.

The gas sensing properties of the elements were measured using a flow-type gas sensing measurement apparatus, as shown in Figure 1. The apparatus was equipped with temperature-controlled water bubbler. Synthetic air was flowed into water bubbler before flowing into the apparatus. The relative humidity of the synthetic air was set at 25, 50, and 75% at 20 °C by controlling the temperature of water bubbler. The elements were placed into quartz tube with internal diameter and length of 4 and 25 cm. The elements were heated at 300 °C using an electric furnace. The original T–VOC test gas (3.66 × 10^4^ μg/m^3^) was diluted with humid air to below 1,000 μg/m^3^ by using mass flow controllers. The heating temperature, 300 °C, was discovered to be the most suitable condition for the Pt,Pd,Au/SnO_2_ and the Pt/SnO_2_ as T–VOC test gas sensors in previous tests. The total flow rate was kept at 200 mL/min. The sensor response (*S*) is defined as Equation (1),

(1) $$S=\frac{R_{a}}{R_{g}}$$

where *R_a_*, and *R_g_* are the electrical resistance in synthetic air and T–VOC test gas, respectively.

## 3. Results

**Figure 2.**
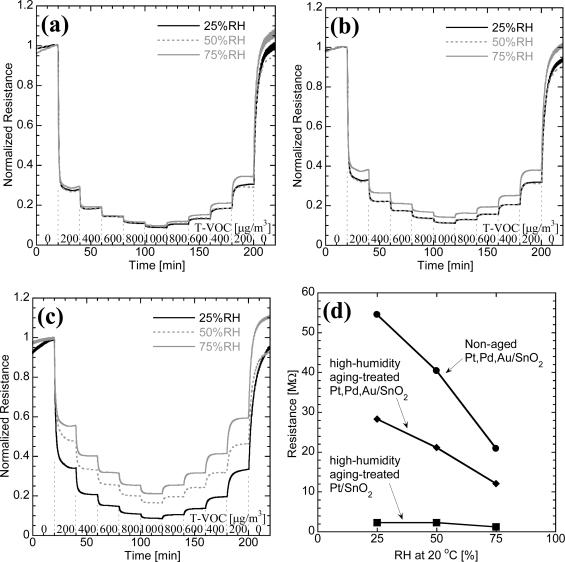
Normalized resistance profiles of (a) high-humidity aged Pt,Pd,Au/SnO_2_, (b) non-aged Pt,Pd,Au/SnO_2_, and (c) high-humidity aged Pt/SnO_2_ in humid air with several concentrations of T–VOC test gas at 300 °C. The black, gray-dashed, and gray lines indicate the normalized resistance of elements in 25, 50, and 75%RH at 20 °C, respectively. The normalization of resistance is based on the resistance at 20 min. Panel (d) shows the base resistance for the normalization, *i.e.*, the real resistances of all elements in 0 μg/m^3^ of T–VOC test gas condition at 20 min in Figure 2a, 2b and 2c.

Figure 2 shows the dynamic resistance responses of high-humidity aged Pt,Pd,Au/SnO_2_ and Pt/SnO_2_ and non-aged Pt,Pd,Au/SnO_2_ thick film elements to the T–VOC test gas at 25, 50, and 75%RH. The resistance of the elements is normalized at the initial change of gas flow from synthetic air to test gas. It can be seen that all elements exhibit distinct responses to the T–VOC test gas. The resistance of all the elements decreased and increased relative to the T–VOC test gas concentration. The resistance changes are almost saturated within 10 min for each step of the gas concentration change. For the aged Pt,Pd,Au/SnO_2_, the decrease in normalized resistance by the T–VOC test gas does not change significantly when the humidity is increased. Thus, the profiles of the aged Pt,Pd,Au/SnO_2_ in Figure 2a are almost identical. For the non-aged Pt,Pd,Au/SnO_2_ (Figure 2b), the response to the T–VOC test gas is almost identical at 25 and 50%RH, whereas it is reduced significantly at 75%RH. However, the drop in resistance of the non-aged Pt,Pd,Au/SnO_2_ to the T–VOC test gas was less severe at 75%RH. It should be noted that the high-humidity aging treated Pt/SnO_2_ element exhibits a lager humidity dependence of the resistance change compared with that of the non-aged Pt, Pd, Au/SnO_2_ elements.

It should be noted that the high-humidity aged Pt/SnO_2_ element exhibits a lager humidity dependence of the resistance change compared with that of the non-aged Pt,Pd,Au/SnO_2_ elements, as shown in Figure 2c. The resistance values of all the elements in the humid synthetic air decrease as humidity increases, as shown in Figure 2d.

**Figure 3.**
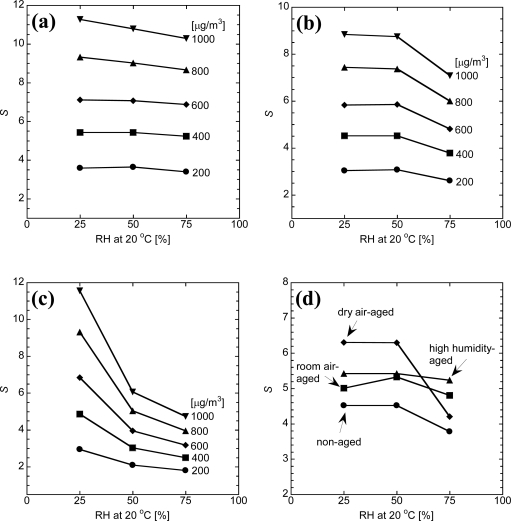
Sensor response (*S*) of (a) high-humidity aged Pt,Pd,Au/SnO_2_, (b) non-aged Pt,Pd,Au/SnO_2_, (c) high-humidity aged Pt/SnO_2_ under several humidity and T–VOC test gas concentration; (d) non, dry air, room air, and high-humidity aged Pt,Pd,Au/SnO_2_ under several humidity and 400 μg/m^3^ T–VOC test gas concentration.

The sensor response (*S*) of all the elements to the T–VOC test gas under different humidity conditions is summarized in Figure 3. The sensor response of the aged Pt,Pd,Au/SnO_2_ element is almost independent of humidity, as shown in Figure 3a. The plots of Figure 3b show that the sensor response of non-aged Pt,Pd,Au/SnO_2_ element to 1,000 μg/m^3^ T–VOC test gas at 75%RH is smaller than that to 800 μg/m^3^ T–VOC test gas at 25 and 50%RH. Thus, the *S* value of the non-aged Pt,Pd,Au/SnO_2_ element depends on humidity, especially under high humidity conditions. For the aged Pt/SnO_2_ element, the sensor response has almost the same performance as the Pt,Pd,Au/SnO_2_ elements at 25%RH. However, the sensor performance is reduced drastically by increasing the relative humidity to higher than 50%RH. The degradation of the sensor performance on the aged Pt/SnO_2_ is almost the same as non-aged Pt/SnO_2_ [7]. The *S* values of room air (approximately 40%RH) and dry air aged Pt,Pd,Au/SnO_2_ elements are compared with those of the high-humidity and the non-aged Pt,Pd,Au/SnO_2_ elements in Figure 3d. Though the sensor performance of the room air-aged Pt,Pd,Au/SnO_2_ element is slightly reduced by relative humidity higher than 50%RH. However, the sensor performance of the dry air-aged Pt,Pd,Au/SnO_2_ element is also reduced drastically by increasing relative humidity to higher than 50%RH. Consequently, these results prove that aging in humidity condition is an effective treatment for reducing the humidity dependency for the Pt,Pd,Au/SnO_2_ but not effective for the Pt/SnO_2_.

## 4. Discussion

The decrease in resistance of the elements is attributed to the combustion of VOCs on the surface of the SnO_2_ grains. In the case of tin oxide, an n-type semiconductor, the oxygen adsorbed on the surface removes electrons from SnO_2_ conduction band at elevated temperatures, producing an electron-depleted layer (space-charge region) [9–11]. The space-charge layer works as a potential barrier between neighboring grains. A reducing gas such as a VOC is oxidized on the surface with consumption of the oxygen adsorbates. During this reaction, the electrons back into the conduction band, resulting in a decrease in the depth of the space-charge region and a drop in resistance.

The resistance decrease with humidity (Figure 2d) should be caused by the adsorption of water molecules which disturbs adsorption of oxygen on the SnO_2_ surface. Therefore, the sensor response of the SnO_2_-based elements generally depends on humidity and it has been confirmed that the combustion on the surface of SnO_2_ is disturbed by moisture [12]. We have shown in our previous report that Pt and/or Au-loading, and Pd-loading act as chemical and electronic sensitization effects, respectively [7]. For the chemical sensitization, the loaded Pt and Au assist the combustion of the T–VOC test gas on the SnO_2_ surface. This effect becomes small under high humidity conditions because the amount of adsorbed oxygen on the SnO_2_ is decreased by coexisting water molecules. For the electric sensitization, the oxidation of the loaded Pd affects the electron-depleted layer [13]. We also have reported that the resistance of Pd-loaded SnO_2_ increased with humidity, whereas that of Pt or Au-loaded SnO_2_ decreased, indicating the advance of the oxidation of loaded Pd surface by the adhered water molecules [7]. In the case of the non-aged Pt,Pd,Au/SnO_2_ element, the immunity of sensor response to humidity change is improved in comparison with SnO_2_, Pt/SnO_2_ and Au/SnO_2_ elements at humidities up to 50%RH. The decrease of the chemical sensitization and the increase of the electronic sensitization are caused simultaneously under high humidity [7]. The high-humidity aging improves the immunity to humidity change for the Pt,Pd,Au/SnO_2_ element but it does not work for the Pt/SnO_2_ element, indicating that the addition of Pd is crucial for realizing the effects of high-humidity aging. The high-humidity aging is found to emphasize the function of the Pd-loaded in the SnO_2_ based elements.

Katti *et al.* have reported XPS patterns of a SnO_2_, an as-prepared 5 wt% Pd-loaded SnO_2_, and a 100%RH-exposed 5 wt% Pd-loaded SnO_2_ at working temperature [14]. Brun *et al.* and Kwoka *et al.* have reported the assignment of XPS O 1s and Sn 3d regions on PdO [15] and SnO_2_ [16,17], respectively. On the basis of O 1s and Sn 3d of the XPS spectra on these reports, the high-humidity exposure induces the oxidation of surface Sn^2+^ to Sn^4+^ and the adsorption of oxygen species on the Pd-loaded SnO_2_ surface [12–15]. For oxidation of surface Sn^2+^ to Sn^4+^, the high-humidity exposure is expected to accelerate to form PdO, whose electron accepter properties transform surface Sn^2+^ to Sn^4+^. The adsorption of oxygen species is assumed to be in the form of water molecule absorbates.

**Figure 4.**
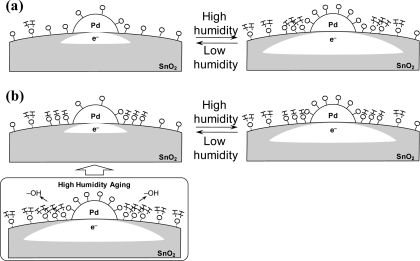
Depictions of the surface conditions of Pd-loading area on (a) the non-aged Pt,Pd,Au/SnO_2_ and (b) the high-humidity aging treated Pt,Pd,Au/SnO_2_.

Here, we propose a high-humidity aging mechanism to improve the immunity to humidity changes. Figure 4a shows the surface conditions around the loaded Pd of the non-aged Pt,Pd,Au/SnO_2_. There are adsorption sites on the SnO_2_ surface where the reversible absorption of oxygen absorbates and water molecules occurs. Under high humidity conditions, the number of absorbed water molecules should be increased compared with that in a low humidity condition, resulting in the decrease of resistance vales and the reduction of response. In the case of the high-humidity aged Pt,Pd,Au/SnO_2_, heat treatment under the high-humidity condition promotes the adsorption of water molecules and the formation of bonding between SnO_2_ and hydroxyl groups derived from the adsorbed water. Because the hydroxyl groups have an electron donating properties [18,19], their formation is dominant around the loaded Pd which acts as an electron acceptor. The formation of the tightly bound hydroxyl group results in the reduction of the reversible absorption sites of water molecules as well as the oxygen absorbates (Figure 4b). The resistance of the high-humidity aged Pt,Pd,Au/SnO_2_ is therefore lower than the non-aged Pt,Pd,Au/SnO_2_, as shown in Figure 2d. The formation of the hydroxyl groups reduces the competing absorption between oxygen and water molecules, indicating the improvement on the immunity to humidity change. The loaded Pd plays an important role in the above described mechanism, so that we observed no effects of high-humidity aging on the Pt/SnO_2_ elements without Pd.

## 5. Conclusions

The sensor response of the non-aged Pt,Pd,Au/SnO_2_ depends slightly on humidity conditions, specifically higher humidity. The high-humidity aging improves the immunity to humidity for the sensor responses of the Pt,Pd,Au/SnO_2_ systems, but is not effective for the Pt/SnO_2_ one. The high-humidity aging-treated Pt, Pd, Au/SnO_2_ elements show responses to the T-VOC test gas which are independent of humidity from 25 to 75 %RH. The loaded Pd plays an important role to realize the immunity to humidity change, and we propose a mechanism for the effects of the high-humidity aging on the basis of this phenomenon.

## Figures and Tables

**Table 1.** Concentrations of each component in the T–VOC test gas [7].

Component	Concentration [ppm]
Acetaldehyde	0.93
Propionaldehyde	0.22
*n*-Butylaldehyde	0.50
*n*-Decane	0.13
Benzene	0.20
Toluene	0.63
*m*-Xylene	0.17
1,2,4-Trimethylbenzene	0.17
Ethylbenzene	0.14
p-Dichlorobenzene	1.14
Ethyl acetate	1.09
Butyl acetate	0.69
Ethanol	1.60
2-Propanol	0.12
Methyl isobutyl ketone (4-methyl-2-pentanone)	0.20
Acetone	1.75
Methyl ethyl ketone (2-butanone)	0.58

Total	10.29*

* 10.29 [ppm] = 3.66 × 10^4^ [μg/m^3^]
